# The Antidiabetic Effect of Grape Pomace Polysaccharide-Polyphenol Complexes

**DOI:** 10.3390/nu13124495

**Published:** 2021-12-15

**Authors:** Filipa Campos, Andreia F. Peixoto, Pedro A. R. Fernandes, Manuel A. Coimbra, Nuno Mateus, Victor de Freitas, Iva Fernandes, Ana Fernandes

**Affiliations:** 1 REQUIMTE—Laboratório Associado Para a Química Verde, Departamento de Química e Bioquímica, Faculdade de Ciências, Universidade do Porto, Rua do Campo Alegre 687, 4169-007 Porto, Portugal; filipasc96@hotmail.com (F.C.); andreia.peixoto@fc.up.pt (A.F.P.); nbmateus@fc.up.pt (N.M.); vfreitas@fc.up.pt (V.d.F.); iva.fernandes@fc.up.pt (I.F.); 2 LAQV/REQUIMTE, REQUIMTE—Laboratório Associado Para a Química Verde, Departmento de Química, Universidade de Aveiro, Campus Universitário de Santiago, 3810-193 Aveiro, Portugal; pedroantonio@ua.pt (P.A.R.F.); mac@ua.pt (M.A.C.)

**Keywords:** anthocyanins, diabetes mellitus, polyphenols, polysaccharides, polysaccharide–polyphenol conjugates, pressurized hot water extraction

## Abstract

Type 2 diabetes mellitus (T2DM) is one of the most prevalent chronic metabolic diseases of the 21st century. Nevertheless, its prevalence might be attenuated by taking advantage of bioactive compounds commonly found in fruits and vegetables. This work is focused on the recovery of polyphenols and polysaccharide–polyphenol conjugates from grape pomace for T2DM management and prevention. Bioactives were extracted by solid–liquid extraction and by pressurized hot water extraction (PHWE). Polyphenolic fraction recovered by PHWE showed the highest value for total phenolic content (427 μg GAE.mg^−1^), mainly anthocyanins and proanthocyanidins, and higher antioxidant activity compared to the fraction recovered by solid–liquid extraction. Polysaccharide–polyphenol conjugates comprehended pectic polysaccharides to which approximately 108 μg GAE of phenolic compounds (per mg fraction) were estimated to be bound. Polyphenols and polysaccharide–polyphenol conjugates exhibited distinct antidiabetic effects, depending on the extraction methodologies employed. Extracts were particularly relevant in the inhibition of a-glucosidase activity, with free polyphenols showing an IC_50_ of 0.47 μg.mL^−1^ while conjugates showed an IC_50_ of 2.7, 4.0 and 5.2 μg.mL^−1^ (solid–liquid extraction, PHWE at 95 and 120 °C, respectively). Antiglycation effect was more pronounced for free polyphenols recovered by PHWE, while the attenuation of glucose uptake by Caco-2 monolayers was more efficient for conjugates obtained by PHWE. The antidiabetic effect of grape pomace bioactives opens new opportunities for the exploitation of these agri-food wastes in food nutrition, the next step towards reaching a circular economy in grape products.

## 1. Introduction

The rapid progress of civilization and lifestyle changes has created factors with adverse effects for the health of society. This has led to increased morbidity from several chronic noncommunicable diseases. Diabetes mellitus (DM) is a serious multifactorial metabolic disorder which has great impact on health and life expectancy of affected individuals. It is characterized by high sugar concentrations in the blood, due to impaired insulin secretion, resistance to peripheral actions of insulin, or both [1]. In the past years, research has been driven into the search of functional bioactive compounds present in plants, namely polyphenols, for type 2 DM (T2DM) prevention and management [2,3]. Indeed, due to their antioxidant properties, polyphenols have gained significant importance in the fields of food nutrition and health. For instance, the role of polyphenols on the inhibition of pro-inflammatory transcription factors or the use of polyphenols as attenuating agents of immune reactions to food by interacting with specific allergen proteins, emphasize the concept of polyphenols as functional ingredients with preventive and therapeutic potential in noncommunicable diseases [4].

In plant-derived foods, polyphenols can be found in free and bound form. While free polyphenols are easily extracted with polar aqueous/organic solvents, bound or non-extractable polyphenols remain insoluble in the solvent used for the extraction, as they are retained in the plant matrix. Non-extractable polyphenols include both high molecular weight polymeric polyphenols (e.g., condensed and hydrolyzable tannins) and low molecular weight phenolic compounds such as phenolic acids and subclasses of some flavonoids, which are associated with cell wall components such as polysaccharides and proteins [5,6,7]. The formation of polysaccharide–polyphenol conjugates may be mediated by several non-covalent interactions such as hydrogen bonding, hydrophobic interaction, or encapsulation within hydrophobic pockets [8,9]. Additionally, polysaccharides may establish covalent bonds with polyphenols as a result of biosynthetic procedures [10,11] or by means of polyphenol oxidation reactions during fruit processing [12].

Polysaccharide–polyphenol conjugates have attracted considerable attention from the scientific community, as they may combine the antioxidant activity of polyphenolic compounds and the physiological effects of polysaccharides [13]. Similar to polyphenols, the consumption of polysaccharides is associated with human health benefits, such as the improvement in gastrointestinal health and the treatment of some cardiovascular diseases and some types of cancer [14]. A reduction in hyperlipidemia, hypertension, modification of the glucose tolerance and insulin response, and increased satiety are other physiological effects associated with the consumption of polysaccharides [15]. Thus, polysaccharide–polyphenol conjugates are certain to have a bright prospect in the fields of functional foods.

Besides fruit and vegetables, agri-food by-products may be a relevant source of bioactive compounds [5]. Grape pomace (*Vitis vinifera* L.), one of the main wastes from the wine industry, can be regarded as an excellent and affordable source of polyphenols, mainly anthocyanins (malvidin-3-*O*-glucoside, peonidin-3-*O*-glucoside, acylated derivatives), flavan-3-ols (epicatechin-3-*O*-gallate and epicatechin, catechins), flavonols (quercetin, myricetin), phenolic acids (gallic acid, syringic acid) and stilbenes (resveratrol) [16]. However, studies regarding the macromolecular fraction of grape pomace are scarce, especially related to polysaccharide–polyphenol conjugates extraction and structural characterization. Concerning polyphenols–wine polymeric material interaction, Gonçalves et al. [17] suggested that they may occur in different energetic layers, ruled by non-covalent interactions and by covalent linkages. Therefore, the research on polysaccharides–polyphenols conjugates, their physicochemical characteristics and bioactivities is crucial for the development of dietary supplements and functional foods.

The recovery of free polyphenols and polysaccharide–polyphenol conjugates can be a solution to mitigate grape pomace as an agri-food disposable. In this work, it was intended to recover free polyphenols and polysaccharide–polyphenol conjugates through solid–liquid extraction and under pressurized hot water conditions. The use of water under these conditions provides advantages over conventional extraction methods, such as being sustainable, faster, and more efficient [18,19,20]. Free polyphenols are well recognized as potent antioxidants and inhibitors of carbohydrate metabolizing enzymes. In this sense, polyphenols bound to polysaccharides may also play a fundamental role as antidiabetic agents. The potential application of these bioactives was evaluated through the modulation of: (i) the activity of carbohydrate-metabolizing enzymes, (ii) the formation of advanced glycation end products (AGEs) and (iii) the intestinal glucose uptake.

## 2. Materials and Methods

### 2.1. Plant Material

Polyphenols and polysaccharide–polyphenol conjugates were extracted from destemmed red grape pomace from Portuguese varieties (*Vitis vinifera* L. cv.), kindly provided by a local winemaker (cultivars located in Chaves, Portugal). Red grape pomace was collected after the last alcoholic fermentation step (7 days) in October 2020, frozen at −20 °C and freeze-dried prior to their use. Prior to the extraction procedure, most of the grape seeds were manually removed.

### 2.2. Extraction Procedures

Polyphenol’s extraction from the grape pomace was performed for 2 h at room temperature using acidified 50% hydroethanolic solutions (pH 2.3 with citric acid at 73% *w/w*) at a ratio of 1:10. The obtained suspension was then filtered on a cloth filter, followed by centrifugation at 10,000 rpm (Dynamica Velocity 14R Refrigerated Centrifuge, Dynamica Scientific Ltd.; Livingston, United Kingdom) for 10 min at room temperature. Organic solvent was removed by evaporation and the aqueous extract was purified by C-18 reversed phase silica gel chromatography using a Büchner funnel and a vacuum filtration system. Briefly, the extract was applied on the top of the gel. Elution was first made with distilled water to remove inorganic salts and small sugars and then with methanol to recover free polyphenols. This fraction was evaporated and freeze dried, giving a free polyphenolic fraction (FP).

Polysaccharide–polyphenol conjugates were obtained by alkaline and pressurized hot water extractions following the general procedure described for medicinal and edible plants [21,22]. Alkaline extraction was performed by suspending 5 g of grape pomace in 50 mL of 0.1 M NaOH for 24 h at room temperature, without any pre-treatment [23]. Thereafter the mixture was extracted at 96 °C under reflux for 6 h. The supernatant was then recovered by centrifugation at 10,000 rpm for 10 min at room temperature (Dynamica Velocity 14R Refrigerated Centrifuge, Dynamica Scientific Ltd.; Livingston, United Kingdom) and neutralized with HCl 0.1 M, giving a crude extract. The isolated crude extract was purified according to Pawlaczyk-Graja and co-workers [24]. First, the crude extract was dissolved in 250 mL of distilled water and extracted twice with *n*-hexane (1:1 *v/v*) for 6 h at 69 °C and with diethyl ether (1:1 *v/v*) for 6 h at 34 °C to remove hydrophobic compounds. The aqueous fraction was evaporated to a paste-like form and treated with 100 mL of methanol at room temperature for 24 h to eliminate low-molecular-weight phenolic compounds [25]. The formed precipitates were recovered by centrifugation at 10,000 rpm for 10 min (Dynamica Velocity 14R Refrigerated Centrifuge, Dynamica Scientific Ltd.; Livingston, United Kingdom) and dried overnight at room temperature. The dried material was redissolved in 150 mL in deionized water, dialyzed for 48 h with at least 6 water renewal, (Spectra/Por^®^, 12–14 MWCO) and freeze dried, giving an alkaline extract (AE).

PHWE was conducted on a Parr Series 4560 Reactor (Parr Instrument Company, Moline, IL, USA), connected to the Parr 4848 Reactor Controller. The extractions were performed using 10 g of grape pomace and 100 mL of distilled water (acidified at pH 2.3 with citric acid at 73% *w/w*) at 120 °C for 30 min. To limit the thermal degradation of polyphenols, a lower extraction temperature (95 °C) and pomace:water ratio was also tested (1:30) for 30 min [26]. Both extractions were performed under inert atmosphere at 5.5 Bar and at 145 rpm. After extraction, the system was cooled down and the extracts were filtered under vacuum, through a glass fiber filter (Whatman GF/C) and centrifuged at 10,000 rpm for 10 min at room temperature (Dynamica Velocity 14R Refrigerated Centrifuge, Dynamica Scientific Ltd.; Livingston, United Kingdom). The extracts were evaporated to a paste-like form and treated as previously described, giving two PHWE extracts, PHWE-95 and PHWE-120.

For the PHWE at 95 °C, the supernatant recovered after precipitation with methanol, was treated to isolate free polyphenols. Methanolic extract was purified by C-18 reversed phase silica gel chromatography as previously described. This fraction was evaporated and freeze dried, giving a free polyphenolic fraction (FP-95).

### 2.3. Physicochemical Characterization

#### 2.3.1. Determination of Polyphenols, Proteins and Sugar Content

Polyphenolic content of each fraction (FP, FP-95, AE, PHWE-95 and PHWE-120,) was determined through the Folin–Ciocalteu assay [27]. Results were expressed as μg gallic acid equivalents (GAE).mg^−1^ dry weight in each fraction.

Protein content was determined using a dye binding assay (Bradford Protein Assay Kit—Thermo Scientific, Waltham, MA, USA), using BSA as standard [28]. Results were expressed as μg BSA equivalents mg^−1^.dry weight in each fraction.

Sugar content in FP and FP-95 extracts was determined by GC-MS after derivatization [29]. Derivatization with hexamethyldisilazane (HMDS) was applied for the trimethylslylation (TMS) of polar functional groups. The procedure was as follows: (a) 600 µL of HMDS:acetonitrile mixture (1:1 *v/v*) was added as a silylation agent to 20 µL of aqueous solution for the derivatization of easily silylable functional groups (e.g., hydroxyl in glucose), 2 µL of trifluoroacetic acid was added as a catalyst and the sample was heated to 50 °C for 30 min. The vial was left open during this process to ensure the escape of the ammonia gas produced in the reaction. Subsequently, (b) in the second step, 400 µL of pure HMDS was added and the mixture was heated to 80 °C for 30 min in a closed vial. After cooling to laboratory temperature 1µL of the resulting solution was injected into the GC–MS/MS system. Derivatization was performed in triplicate. The GC–MS/MS analyses were carried out with a Trace 1300 gas chromatograph equipped with a split–splitless injector, an autosampler 1310 Thermo Scientific and a ISQ Single quadrupole MS (Thermo Fisher, Austin, TX, USA). A total of 1 µL of the sample was injected into the injector operating in splitless mode. The temperatures of the injector and the MS-transfer line were 250 °C and 300 °C, respectively. Compounds were separated on a 30 m × 0.25 mm (i.d.) × 0.25 µm DB-17 capillary column (Agilent Technologies, CA, USA) operating at constant helium flow of 1.5 mL.min^−1^. The column temperature was initially set to 110 °C, held for 5 min, increasing then at a rate of 6 °C.min^−1^ to 300 °C and held for 5 min. Measurements were performed in SCAN mode with m/z range set to 40–1100. The MS conditions were as follows: ion source temperature 280 °C and electron energy 70 eV, using glucose as standard for the calibration curve. Selected ion monitoring (SIM) conditions were used for the glucose, selecting the *m/z* 204. Results were expressed as μg glucose equivalents mg^−1^.dry weight in each fraction.

#### 2.3.2. Carbohydrate Analysis

The carbohydrate compositions of AE, PHWE-95 and PHWE-120 extracts were determined by neutral sugars and uronic acid quantification. Neutral sugars were quantified by GC-FID after acid hydrolysis and derivatization to alditol acetates, using deoxyglucose as internal standard [30]. Uronic acids (UA) were quantified by the 3-phenylphenol colorimetric method [31] using d-galacturonic acid as a standard. Results were expressed as mg sugar.g^−1^ dry sample.

#### 2.3.3. Polymeric Colour Index

Polymeric colour index was determined by measuring the absorbances of FP and FP-95 extract solutions that had been treated with sodium bisulfite (20%) compared to non-treated samples [32]. 2.8 mL of diluted samples (in distilled water) were transferred to each of two cuvettes, and 0.2 mL of bisulfite solution or distilled water was added. The samples were left to equilibrate in the dark for 15 min. The absorbance of each sample was measured at 420 nm, λ_max_ and 700 nm (to correct for haze) on a UV-Visible spectrophotometer (Thermo Scientific Evolution Array, Waltham, MA, USA). The colour density of the control sample (treated with water) and polymeric colour (bisulfite bleached sample) was calculated as follows:

Colour Density = [(A420 nm − A700 nm) + (Aλmax − A700 nm)] × DF

Polymeric color = [(A_420 nm_ − A_700 nm_) + (A_λmax_ − A_700 nm_)] × DF

where DF is the dilution factor.

The percentage of polymeric colour was calculated using the formula:

Percentage polymeric color = (polymeric color/color density) × 100

#### 2.3.4. Reverse Phase Liquid Chromatography Analysis of Polyphenols

The anthocyanins content in FP and FP-95 extracts was analyzed by UPLC-DAD (Dionex Ultimate 3000, Thermo Scientific, Waltham, MA, USA) on a C-18 gel column (250 mm × 4.6 mm i.d.; 5 μm, Thermo Scientific), using HCOOH/H_2_O (10/90, *v/v*) and HCOOH/CH_3_CN/H_2_O (10/30/60, *v/v*) as solvents [33]. Results were expressed as μg malvidin-3-*O*-glucoside equivalents.mg^−1^ dry weight.

To analyze the content of non-anthocyanic compounds (low molecular weight polyphenols, proanthocyanidins and flavonols), aqueous extracts were mixed with ethyl acetate and acetonitrile 2/2/1 (*v/v/v*) in microtubes, placed in a shaker for 10 s and centrifuged for 5 min at 8000 rpm. After centrifugation and phase separation, liquid–liquid extraction was repeated for the aqueous phase. Organic phases were combined, and the organic solvent evaporated in a speed vacuum [34]. The obtained fraction was then re-suspended in water/methanol (1/1; *v/v*) and analyzed by UPLC-DAD, using CH_3_COOH/H_2_O (1/99, *v/v*) and CH_3_COOH/CH_3_CN/H_2_O (1/20/79, *v/v*) as solvents [35]. Results were expressed as μg GAE.mg^−1^ dry weight.

#### 2.3.5. High Performance Liquid Chromatography-Mass Spectrometry (HPLC-DAD/ESI-MS) Analysis of Polyphenols

The identification of polyphenols was performed by LC-DAD/ESI-MS [35,36,37]. A Finnigan Surveyor series liquid chromatograph equipped with a Thermo Finnigan (Hypersil Gold) C-18 reversed-phase column (150 mm × 4.6 mm, i.d.; 5 μm, Thermo Scientific) thermostatted at 25 °C was used. Detection was carried out between 200 and 700 nm using a Finnigan Surveyor PDA Plus detector. Mass detection was made on a Finnigan LCQ DECA XP MAX (Finnigan Corp., San Jose, CA, USA) quadrupole ion trap equipped with an atmospheric pressure ionization (API) source using an electrospray ionization (ESI) source. The vaporizer and capillary voltages were 5 kV and 4 V, respectively. The capillary temperature was set at 325 °C. Nitrogen was used as both sheath and auxiliary gas at flow rates of 80 and 30, respectively (in arbitrary units). Spectra were recorded in the negative- or positive-ion mode between *m*/*z* 120 and 2000. The mass spectrometer was programmed to do a series of three scans: a full mass, a zoom scan of the most intense ion in the first scan, and an MS–MS of the most intense ion using relative collision energies of 30 and 60 V.

To identify the polyphenolic compounds, present in the conjugate fractions, these fractions were hydrolyzed with an acidic solvent [38,39,40]. Briefly, 20 mg of conjugates were dissolved in 1.5 mL of distilled water and hydrolyzed by adding 0.5 mL of HCl (37% *w/w*). The mixture was incubated in thermoblock at 85 °C for 30 min. The aqueous fraction was extracted with 1.5 mL of diethyl ether:ethyl acetate (1:1 *v/v*). The mixture was vortexed for 45s and centrifuged for 10 min at 10,000 rpm. This extraction was performed three times. The organic layers containing phenolic acids liberated from acid hydrolysis were combined and evaporated to dryness on a speed vacuum (CentriVac Concentrator Labconco, Kansas City, MO, USA) while the aqueous layer was freeze-dried. Residues were re-dissolved in 0.100 mL water and samples were analyzed by reversed-phase UPLC-DAD and LC-DAD/ESI-MS as described in the Section 2.3.4.

### 2.4. Antioxidant Activity

Ferric Reducing Antioxidant Power (FRAP) and antiradical activity, using 2,2′-diphenyl-1-picrylhydrazyl radical (DPPH) assays, were performed according to the literature [41,42] with some modifications. FRAP solution consisted of a mixture of 1 mL of TPTZ, 1 mL of iron (III) chloride and 10 mL of acetate buffer (300 mmol.L^−1^, pH 3.6), placed in the oven at 37 °C for 10 min; 10 mL of this mixture were diluted in 20 mL of acetate buffer. In 96-well plates, 270 µL of FRAP solution and 30 µL of aqueous solution of FP, FP-95, AE, PHWE-95 and PHWE-120 extracts were mixed, and the absorbance at 593 nm at 37 °C was measured at 0 and 4 min on a plate reader (Biotek Powerwave XS, Santa Clara, CA, USA). Results were expressed as μM Trolox equivalents.

For the DPPH assay, in a 96-well plates, 270 µL of DPPH solution (prepared in methanol at a concentration 24.2 µg.mL^−1^) was mixed with 30 µL of aqueous solution of FP, FP-95, AE, PHWE-95 and PHWE-120 extracts and the absorbance at 515 nm was recorded every 5 min for 20 min on a plate reader (Biotek Powerwave XS, Santa Clara, CA, USA). Results were expressed as μM Trolox equivalents.

### 2.5. Antidiabetic Properties

#### 2.5.1. α-Amylase and a-Glucosidase Inhibitory Assay

The inhibitory activity of free polyphenols extracts (FP, FP-95) and polysaccharide–polyphenol conjugates (AE, PHWE-95 and PHWE-120) extracts was conducted with pancreatic porcine α-amylase (A6255, Sigma Aldrich) and α-glucosidase from Saccharomyces cerevisiae (G5003, Sigma Aldrich) using ethylidene-4-nitrophenyl-α-D-maltoheptaoside and 4-nitrophenyl-α-D-glucopyranoside as enzymes substrates (for α-amylase and α-glucosidase, respectively). All solutions were prepared in 10 mM phosphate buffer saline solution (pH 6.8), except for FP and FP-95 which were prepared in DMSO. First, α-amylase (15 U.mg^−1^) or a-glucosidase (0.011 U.mg^−1^), alone or with different concentrations of each fraction, was pre-incubated at 37 °C for 10 min. Then, substrate at 2.5 mM or 0.269 mM (a-amylase and a-glucosidase, respectively) was added to the reaction mixture and the reaction was followed for 50 min at 405 nm and 37 °C on a plate reader (FlexStation 3 Multi-Mode Microplate Reader). Acarbose, an oligosaccharide of microbial origin that is used as inhibitor of carbohydrate digestion for clinical management of T2DM [43], was used as the positive control. Results were expressed as inhibition percentage (Equation (1)) and a non-linear regression dose–response curve was established to calculate IC_50_ values (µg.mL^−1^) of each fraction.

(1) $$Inhibition\;\%=100-\left(\;\frac{A_{control\;}-A_{sample}}{A_{control}}\times 100\right)\;$$

#### 2.5.2. Advanced Glycation End Products (AGEs) Assay

The AGEs assay uses fluorescence spectroscopy to monitor the inhibitory effect exerted against glycation in the presence or absence of an extract using a reaction model system. The antiglycation activity of polyphenols extracts (FP, FP-95) and polysaccharide–polyphenol conjugates (AE, PHWE-95 and PHWE-120) extracts was determined by BSA-glucose model system [44]. Briefly, 5.0 mL of reaction mixture (5% BSA (w/v), 500 mM glucose and 0.1% sodium azide dissolved in phosphate buffer (200 mM, pH 7.4)) and polyphenols and conjugates fractions at different concentrations were prepared. Aminoguanidine (AG) was used as the positive control. A blank was prepared without positive control or extracts. The tubes were caped and incubated for 14 days at 37 °C in the dark in a temperature-controlled incubator. The fluorescence of the glycated solution was determined using a spectrofluorometer at an excitation/emission wavelength of 370/440 nm, which is characteristic of advanced glycation end products (AGEs). Results were expressed as inhibition percentage on AGE (Equation (1)) and a non-linear regression dose–response curve was established to calculate the IC_50_ values (µg.mL^−1^) of each fraction.

#### 2.5.3. Glucose Uptake Assay

C2BBe1 (CRL-2102, ATCC) [clone of Caco-2] intestinal cell culture was selected on the basis of morphological homogeneity and exclusive apical villin localization. C2BBe1 cells form a polarized monolayer with an apical brush border morphologically comparable to that of the human colon. Transport study was performed according to the previous published method [45]. C2BBe1 cells were grown at 37 °C in an atmosphere of 5% CO_2_ and 95% relative humidity and were cultured in Minimum Essential Medium Eagle (MEME) that was supplemented with 15% fetal bovine serum, 25 mM HEPES, 1% Glutamax, 1% sodium pyruvate 100 Units.mL^−1^ penicillin, 100 mg.mL^−1^ streptomycin, and 0.25 mg.mL^−1^ amphotericin B (all from Sigma). Culture medium was changed every 2 days, and the cells were passed at the split ratio of 1:4 after full confluence. For transport experiments, C2BBe1 cells were seeded on Transwell inserts at 1.5 × 10^5^ cells.mL^−1^ (polycarbonate membrane, 0.4 µm pore size, 24 mm diameter, Corning, New York, NY, USA). After 21 days, cell monolayers were formed and differentiated. Trans-epithelial electrical resistance values (TEER) in each well were measured using Millicell epithelial voltommeter (Millipore Co., Bedford, MA, USA) with “chopstick” electrodes. Only cell monolayers with TEER values higher than 230 Ω were used for the experiments. Then, the medium was removed, and cells were washed with Hanks buffer (HBSS) (pH 7.4). A total of 0.5 mL of HBSS with glucose (25 mM, simulated fed state) in the presence or absence of either polyphenols (FP-95) (0.26 mg.mL^−1^) or polysaccharide–polyphenol conjugates 95 °C (PHWE-95) (10 mg.mL^−1^) were added to the apical side of the wells and 1.5 mL of HBSS without glucose was added to the basolateral side. After 2 h of incubation, samples were collected from the basolateral and apical side and then frozen (−18 °C) until GC-MS analysis of glucose content in them, following the method described in Section 2.3.1. Transport efficiency percentages were calculated according to the following formula: ((compound concentrations at the basolateral side overtime)/(compound concentrations at the apical side at the zero h)) × 100.

Following permeability experiments, the cells were incubated with Lucifer yellow at a concentration of 100 µM for 30 min on the apical side of the Transwell insert. HBSS was collected from the apical and basolateral sides at t = 0 and t = 30 min and analysed by fluorescence at 458 nm/530 nm using a FlexStation 3 Multi-Mode Microplate Reader (Molecular Devices, CA, USA). Lucifer yellow flux range for intact cell monolayers is typically 0.3% to 2% [46]. Thus, cell layers that transported more than 2% of Lucifer yellow to the basolateral compartment were judged as leaking and were discarded.

### 2.6. Statistical Analysis

Experiments were performed in triplicate (3 replicates for each triplicate, *n* = 9, unless otherwise stated) to ensure the reproducibility of the results. Data are expressed as the mean ± standard error of the mean (SEM) or mean ± standard deviation (SD). One-way analysis of variance (one-way ANOVA) was used to determine statistically significant differences between the means of different experimental groups using the Tukey’s multiple comparisons test. Differences were statistically significant at *p* < 0.05. All statistical data were processed using GraphPad Prism version 8.0 for Windows.

## 3. Results and Discussion

### 3.1. Characterization of Grape Pomace Free Polyphenols

Free polyphenols were obtained from grape pomace through a hydroalcoholic solid–liquid extraction (FP) and by pressurized hot water extraction at 95 °C (FP-95). Although these fractions were recovered at comparable yields (1.5% and 1.3% for FP and FP-95, respectively), the FP-95 fraction showed a total phenolic content (427 μg GAE.mg^−1^) higher than the FP fraction (254 μg GAE.mg^−1^). PHWE allows the physicochemical changes of water, decreasing the dielectric constant. The higher temperatures also favour water to wet and penetrate the cell-wall matrix and causes a decrease of the surface tension and viscosity, improving analyte diffusion rate and mass-transfer kinetics. Considering the moderate experimental conditions applied and the use of water, this may be considered a low energy consumption process with no toxicity associated.

The FP-95 fraction also presented the highest antioxidant activity, almost two-fold than the observed for FP, being in line with the highest value of total polyphenols determined in this extract.

UPLC-DAD analysis showed that FP and FP-95 fractions presented similar amounts of anthocyanins (*p* > 0.05), with the dominant anthocyanin being malvidin-3-*O*-glucoside (major red grape anthocyanin) and its acylated derivatives such as malvidin-3-*O*-6″-*p*-coumaroyl-glucoside and malvidin-3-*O*-6″-acetyl-glucoside (Supplementary Materials, Table S1 and Figure S1). Delphinidin-3-*O*-glucoside, peonidin-3-*O*-glucoside and petunidin-3-*O*-glucoside, as well as acylated derivatives (petunidin-3-*O*-6″-acetyl-glucoside, delphinidin-3-*O*-6″-*p*-coumaroyl-glucoside, petunidin-3-*O*-6″-*p*-coumaroyl-glucoside and malvidin-3-*O*-6″-caffeoyl-glucoside), were also detected, but in minor amounts. Anthocyanin-derived pigments such as carboxypyranomalvidin-3-*O*-glucoside, carboxypyranomalvidin-3-*O*-acetyl-glucoside and carboxypyranopetunidin-3-*O*-acetyl-glucoside were also detected. These pigments result from the reaction of anthocyanins with yeast metabolites during the fermentation process [47].

Polymeric anthocyanins can also be formed during grape fermentation from the reaction between monomeric anthocyanins and other phenolic compounds. Acetaldehyde-mediated condensation, copigmentation and self-association [48] are the major reactions considered to be responsible for the formation of polymeric pigments. Thus, the percentage of polymeric colour was determined considering the ratio between polymerized coloured anthocyanins and colour density. This value was much higher for FP extract (about 80%), an indication of a higher proportion of polymeric anthocyanins than for FP-95. Polymeric anthocyanins generally have higher hydrophobicity and larger molecular weight compared to monomeric anthocyanins, thus being preferably extracted by organic solvents. They also typically show poor solubility in acidic aqueous systems, maybe due to the other compounds bound to anthocyanins [49]. In fact, FP solutions presented visible suspensions, even at lower concentrations, suggesting lower solubility of the solutes. FP-95 extract showed a much lower percentage of polymeric colour (30%), indicating the preferential recovery of monomeric anthocyanins.

Besides anthocyanins, free polyphenolic fractions also presented monomeric and oligomeric flavan-3-ols and conjugated phenolic compounds with sugar moieties (Supplementary Materials, Table S2 and Figure S2). Low molecular weight polyphenols, such as gallic acid (*m/z* 169) and coumaric acid-3-*O*-glucoside (*m/z* 325), could be detected in both fraction with these compounds corresponding to minor components (approximately 4 and 5% of the total amount of detected phenolic compounds for FP and FP-95, respectively).

Regarding proanthocyanidins, they were detected in a much higher proportion. The LC-DAD/ESI-MS analysis allowed to identify monomers (catechin and epicatechin, pseudo-molecular ion *m/z* 289), oligomeric proanthocyanidins (dimers and trimers, pseudo-molecular ion *m/z* 577 and 865, respectively) and gallate derivatives in both fractions (pseudo-molecular ion *m/z* 729 and 881, mono and digallate derivative, respectively). Gallate trimers and tetramers could also be detected in these fractions, but in smaller amounts (pseudo-molecular ion *m/z* 1017 and 1153, respectively). Additionally, the presence of the pseudo-molecular ions at *m/z* 593 and 897 indicates the presence of prodelphinidins in grape pomace. Compound 3, with the pseudo-molecular ion at *m/z* 593 and with characteristic fragments at *m/z* 467, 425 and 407, suggested the presence of a dimeric proanthocyanidin formed with one (epi)gallocatechin unit and one (epi)catechin unit. Compound 4, with the pseudo-molecular ion at *m/z* 897, suggested the presence of a trimeric proanthocyanidin with two (epi)gallocatechin units and one (epi)catechin unit [50,51].

Four flavonols were detected in grape pomace free polyphenolic fractions (8–9% of the total amount of detected phenolic compounds for FP and FP-95, respectively), with quercetin-3-*O*-glucuronide as the major one. Quercetin-3-*O*-hexoside, myricetin-3-*O*-hexoside and myricetin-3-*O*-arabinoside could also be detected, but in lower amounts.

In general, pressurized hot water extraction allowed us to obtain a fraction rich in proanthocyanidins, with minor amounts of low molecular weight phenolics and flavonols, which could be correlated with the highest antioxidant activity observed for this fraction. Besides polyphenols, both fractions were also shown to present similar amounts of proteins (approximately 10%) and simple sugars (<1%).

### 3.2. Characterization of Grape Pomace Polysaccharide–Polyphenol Conjugates

As the physicochemical characteristics of the macromolecular fraction, such as molecular weight, chemical composition, and related bioactivities, have been shown to be affected by the extraction methodology [21,52,53], alkaline and pressurized hot water extractions were tested to recover polysaccharide–polyphenol complexes. A preliminary evaluation of the extracts allowed to observe some physical differences. Although the three fractions presented a fiber structure, the fraction obtained by alkaline extraction (AE) resulted in a fiber structure with a beige colour. On the other hand, conjugates obtained from PHWE (PHWE-95 and PHWE-120) presented a reddish colour (more significant for the lowest temperature), a possible indication of the presence of anthocyanins co-extracted with the fiber matrix.

The high molecular weight material isolated by dialysis accounted for 1.3 to 3.4% (g fraction/g dried grape pomace), with the highest value corresponding to the PHWE-120. This was attributed to the higher temperatures used, which promoted a higher degradation of the cell wall structure, and thus, a higher solubilization of polymeric material than when performing the extraction at 95 °C or alkaline extractions at room temperature (Table 1).

Sugar analysis revealed that the polymeric material of AE, PHWE-95 and PHWE-120 was mostly represented by polysaccharides (61% to 69%), differing on their sugar composition (Table 2). The AE polysaccharides were richer in GalA (79 mol%), a sugar typical of galacturonan chains of pectic polysaccharides found in grape pomace [24]. The polysaccharides of PHWE-95 were also rich in GalA (59 mol%) but presented a higher proportion of neutral sugars such as Ara (20 mol%) and Gal (8 mol%) characteristic of arabinans and galactan side chains of pectic polysaccharides of grape skin cell walls [54,55]. These sugars were also found in the PHWE-120 extract, alongside with Glc (27 mol%), which has been also reported for skin HEPES-soluble polysaccharides [55]. This is the same outcome from the solubilization of xyloglucan degraded material under high temperatures as reported for apple pomace [56].

Alongside with polysaccharides, polyphenols were found to be present in all extracts at a proportion of 106 to 108 and 111 μg GAE equivalents per mg of extract for AE, PHWE-95 and PHWE-120, respectively. These compounds were not removed during the washing step with methanol and after the extensive dialysis, thus suggesting that they are part of a macromolecular structure composed by polysaccharides. In fact, the chaotropic nature of methanol, which causes the cleavage of hydrogen bounds and hydrophobic interactions between polyphenols and polysaccharides [57], might be an indication of the existence of covalently linked polysaccharide–polyphenols complexes, instead of non-covalent complexes resulting from biosynthetic pathways or due to grape pomace processing, as previously observed for apple pomace [39]. Proteins were also found to be present at a proportion of 1% (Table 1).

**Figure 1:**
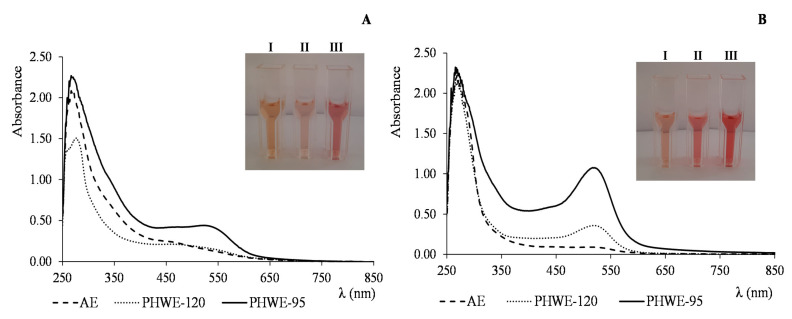
UV-Visible spectra of polysaccharide–polyphenol conjugates fractions (**A**) before acidic hydrolysis and (**B**) after acidic hydrolysis. I, II and III cells correspond to AE, PHWE-120 and PHWE-95 extracts, respectively.

To understand the nature of the polysaccharide–polyphenol conjugates, the UV-Vis spectra of the samples were obtained. Conjugate fractions showed a band around 280 nm which is likely to arise, besides proteins, from the presence of aromatic structures of phenolic compounds (C=C bonds of aromatic rings). Another band with lower intensity at approximately 330 nm was also present in the spectra, typically related with the presence of subunits of phenolic conjugated to each other (C=C bonds of the aromatic structure) [58]. The PHWE extracts also showed a band around 520–530 nm, characteristic of the presence of anthocyanins (Figure 1A). For AE extract, the alkaline extraction performed without NaBH_4_ may have induced polyphenols oxidation and degradation, explaining the lack of this band. However, despite the band around 520–530 nm, no chromatographic peaks could be detected by HPLC-DAD, suggesting, as reported for melanoidins, the covalent linkage of polyphenols to the polymeric material [40], instead of an association mediated by non-covalent interactions. Acid hydrolysis of the polymeric material resulted in an increase in the band around 520 nm (Figure 1B). Additionally, chromatographic peaks could be detected. The MS spectra showed characteristic signals at *m/z* 287, 303 and 331, corresponding to cyanidin, delphinidin and malvidin aglycones, respectively. The presence of cyanidin and delphinidin aglycones could arise as an artefact from the hydrolysis procedure which may have converted proanthocyanidins in anthocyanidins due to acidic hydrolysis at higher temperatures. This agrees with previous results, where polymeric tannins are the most common phenolic compounds linked to fruits dietary fibers [59]. However, the detection of the pseudo-molecular ion *m/z* 331 corresponding to malvidin aglycone shows that besides proanthocyanidins, anthocyanic pigments might also be bound to the polymeric material, especially pectic polysaccharides. The occurrence of these phenolic structures was in accordance with the antioxidant activity observed for all fractions, as measured by the DPPH and FRAP assays.

Regarding the DPPH-radical assay, the PHWE polysaccharide–polyphenol complexes presented a similar scavenging capacity (*p* > 0.05), which was, however, higher than AE polysaccharide–polyphenol complexes (16 ± 3; 27 ± 1 and 25 ± 2 mM Trolox equivalents for AE, PHWE-95 and PHWE-120, respectively). However, no differences (*p* > 0.05) were observed for the FRAP assay (15 ± 2; 12 ± 2 and 16 ± 2, mM Trolox equivalents, for AE, PHWE-95 and PHWE-120, respectively). The differences in the antioxidant capacity of the three conjugates results from the type of polyphenols adsorbed and the extent of carbohydrates able to link or adsorb polyphenols. Globally, these values were noticeably higher than that of the free polyphenol’s extracts (FP and FP-95), possibly due to the cumulative effect of polysaccharides to samples’ weight.

### 3.3. Evaluation of the Potential Antidibetic Effects

#### 3.3.1. Inhibition of α-Amylase and α-Glucosidase Activity

Polyphenols and plant polysaccharides may affect starch polysaccharides digestibility by inhibiting α-amylase and α-glucosidase [60], and consequently, the rate of glucose release in the bloodstream, a feature imbalanced in metabolic disorders and associated diseases. For that reason, the inhibitory activity of grape pomace polyphenols and polysaccharide–polyphenol conjugates on these enzymes was studied and compared to the effect of acarbose (positive control). The inhibition of α-amylase and a-glucosidase was dose-dependent and considerably different among free and polysaccharide bond–polyphenolic fractions (Table 3). Despite the lower amount of polyphenols observed in the extract obtained by 50% ethanol extraction (FP), it presented a similar inhibitory activity as the polyphenolic fraction obtained by pressurized hot water extraction at 95 °C (FP-95). This can be attributed to the presence of polymerized pigments that, as reported for grape seed procyanidins [61], present a higher inhibitory activity when compared to the lower molecular weight counterparts. Also, the presence of larger molecules may confer to these pigments a greater chemical stability in mildly to neutrally aqueous environments, which may induce similar inhibitory activity, despite the lower amount. Free polyphenol inhibition may possibly be due to the capability of free polyphenols, rich in hydroxyl groups, to interfere with substrate–enzyme binding, by blocking the active sites of the enzymes or through the non-specific interactions at different enzymes domains, via hydrogen, hydrophobic or electrostatic interactions. These extracts provided a more pronounced inhibitory effect on α-glucosidase, compared with α-amylase, similarly to the results previously reported. According to the Rasouli et al. (2017) work [62], several polyphenols have shown a high affinity towards α-glucosidase active sites, such as epicatechin, cyanidin, ferulic and caffeic acid, quercetin or syringic acid. Conversely, inhibition of α-amylase activity was achieved by fewer classes of polyphenols. FP and FP-95 extracts were composed mainly of proanthocyanidins, anthocyanins and low molecular weight polyphenols and some flavonols which may explain the higher affinity to α-glucosidase active sites and increased inhibition activity. Additionally, the IC_50_ values observed for a-glucosidase activity, were significantly lower than acarbose (current available drug), a very remarkable aspect related to the potential application of these extracts in the development of functional foods for the management of T2DM.

Polyphenol–polysaccharide conjugates showed an inhibitory effect on α-amylase 20- to 80-fold lower than the free polyphenolic fractions and 10-fold lower for a-glucosidase. The macromolecular nature of polysaccharide–polyphenol conjugates clearly affected polyphenols’ capability to modulate enzyme activity, possibly due to steric hindrance caused by the linkage to polysaccharides, thus resulting in a lower inhibition activity. In fact, if the IC_50_ values for a-amylase inhibition, were expressed in polyphenol concentration (GAE) instead of extract concentration, a higher IC_50_ value could still be observed for the conjugates, suggesting the unfavorable role of the polysaccharide part of the conjugate for the binding (estimated IC_50_ values of 6.25; 11.8; 227; 62; and 103 μg.mL^−1^ for FP, FP-95, AE, PHWE-95 and PHWE-120, respectively).

Additionally, the methodology applied for polysaccharide–polyphenol conjugates extraction, allowed us to obtain fractions with different inhibitory activity towards α-amylase. Oxidized polyphenolic–polysaccharide structures obtained by alkaline extraction resulted in significantly lower inhibition activity. Conversely, non-oxidized polyphenolic structures, obtained by PHWE, showed a significantly higher inhibition activity (lower IC_50_). PHWE-120 extract, with xyloglucan domains and lower amounts of neutral sugars, showed lower inhibition activity compared to PHWE-95 extract, despite similar polyphenolic content. This suggests that branched domains richer in neutral sugars recovered at lower temperatures, promote a higher ability of polyphenols bound to polysaccharides to inhibit a-amylase activity. Regarding a-glucosidase inhibitory activity, an opposite trend could be observed with oxidized polysaccharide–polyphenol structures richer in homogalacturonan domains showing a higher inhibition activity.

#### 3.3.2. Advanced Glycation End Products (AGEs)

The antiglycation activities of free polyphenols and polysaccharide–polyphenol conjugates were investigated and compared to the activity of aminoguanidine (positive control), a common therapeutic drug used to prevent the formation of AGE.

Polyphenol extract obtained by pressurized hot water extraction (FP-95) presented antiglycation activity similar (*p* > 0.05) to aminoguanidine (AG), with an IC_50_ value of 209 μ.mL^−1^ (Table 4). This comparable activity enhances the importance of the inclusion of grape pomace polyphenolic extract in our daily diet. Free polyphenol extract obtained by solid–liquid extraction (FP) presented a lower inhibition activity as shown by the higher IC_50_ value (571 μg.mL^−1^). This could be related to the lower antioxidant activity of this fraction when compared to the FP-95 fraction (Table 1).

For polysaccharide–polyphenol conjugates, the IC_50_ values obtained was 2- to 10-fold higher when compared to polyphenolic fractions, in agreement with the data for the enzymatic inhibition essays. The complexes obtained by alkaline extraction (AE) and PHWE obtained at 95 °C presented a comparable antiglycation activity but lower than that of the complexes obtained by PHWE at 120 °C (PHWE-120). Although polysaccharide–polyphenol fractions generally presented higher antioxidant activity (particularly free radical scavenging activity), the lower antiglycation activity compared to free polyphenols may be due to the prevalence of other mechanisms of action to inhibit glycation and formation of AGE in the polyphenolic extracts. In fact, anthocyanin-3-glucosides or phenolic acids may bind competitively to proteins by non-covalent interactions (hydrogen bonds or van der Waals forces), thus protecting their structural integrity and inhibiting nonenzymatic glycation. Additionally, some phenolic compounds attenuate the expression of AGE receptor-associated signaling, while others may trap active dicarbonyl compounds or chelate metal ions [63,64]. Expressing the antiglycation activity as GAE instead of extract concentration resulted in a similar IC_50_ between free polyphenols and polysaccharide–polyphenols conjugate (estimated IC_50_ values of 145; 89; 110; 137; and 569 μg.mL^−1^ for FP, FP-95, AE, PHWE-95 and PHWE-120, respectively), thus suggesting the reduced impact of polysaccharides for the global antiglycation effect of the conjugates. However, despite the lower impact of polysaccharide moieties, polyphenols associated with macromolecular structures are poorly bioavailable in the upper gut and reach the colon, thus contributing to the target delivery and bioactivity of polyphenols.

#### 3.3.3. Glucose Uptake

To measure glucose uptake, an intestinal epithelial layer of Caco-2 cells was cultured on a Transwell system and glucose transport was measured after 120 min by GC-MS. To validate these experiments, a transport assay with Lucifer yellow was performed. The observed medium % flux (0.55 ± 0.04%), less than 2%, allowed the validation of the experiments [46].

**Figure 2:**
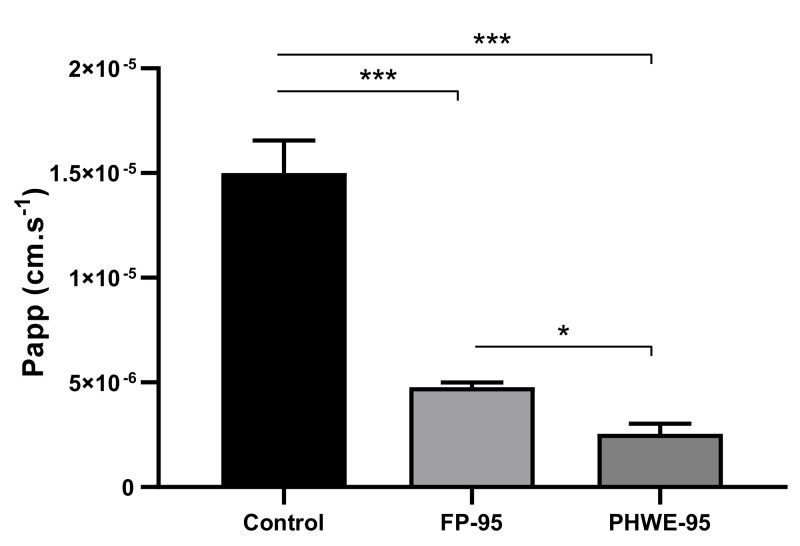
Glucose permeability coefficient (cm.s^−1^) in the presence of a FP-95 (0.26 mg.mL^−1^) or PHWE-95 (10 mg.mL^−1^). In all samples glucose was added to the apical side at 25 mM glucose in order to simulate fed state and basolateral side was filled with Hanks glucose-free buffer. Results are presented as the mean ± standard error of the mean (SEM) (*n* = 8). Significative differences versus control samples *** *p* < 0.001; significative difference between FP-95 and PHWE-95 * *p* < 0.05.

With Hanks buffer containing 25 mM glucose and no polyphenols or polysaccharide–polyphenol conjugates, it was possible to quantify 0.6 mM of glucose in the basolateral side (approximately 8% transported glucose) after 120 min, corresponding to an apparent permeability of 1.50 × 10^−5^ ± 1.55 × 10^−6^ cm.s^−1^ (Figure 2). A similar glucose transport percentage has been previously reported [65]. Glucose transport was found to be impacted by the presence of free polyphenolic fraction (FP-95), with a significative reduction being observed in the uptake of glucose for 4.77 × 10^−6^ ± 2.26 × 10^−7^ cm.s^−1^ (approximately 2.6% transported glucose) after 120 min of incubation (*p* < 0.001). This reduction may be attributed to the presence of glycosylated anthocyanins or flavonols, that inhibit the active glucose transport by sodium-dependent glucose co-transporters 1 (SGLT1) and glucose transporter 2 (GLUT2) by direct competition and also at a gene level [66]. Other polyphenols like proanthocyanidins are also able to inhibit facilitated glucose uptake via steric hindrance [67].

Considering the polysaccharide–polyphenol conjugates, a higher concentration was tested since only 10% of the total amount corresponded to polyphenolic content. In this case, an even lower glucose uptake was observed, suggesting that besides the contribution of polyphenols, other factors were affecting glucose uptake. Also, a significative difference in the apparent coefficient was determined between the two extracts, highlighting for the dual effect of the conjugates (*p* < 0.05) due to their polyphenolic and polysaccharide nature. Some reports have already shown the intestinal uptake of certain types of polysaccharides against Caco-2 monolayer cells [65,68], with this transport being time-dependent and occurring in the form of macromolecules. Additionally, the results presented by Cai et al. (2017) [65] clearly showed that polysaccharides from *Lycium barbarum* L. inhibit glucose transport across the Caco-2 monolayer cells mainly by competing for SGLT1 transporters (to a less extension, by GLUT2 competition) and also by down regulating the expression of SGLT-1. The increased viscosity at high polysaccharide concentration, may also impair glucose diffusion and transport.

## 4. Conclusions

Polyphenols, either as free or conjugated with polysaccharides, were isolated from grape pomace. PHWE was shown to be a suitable methodology, allowing us to obtain both bioactives in good yields without the use of organic solvents. Polyphenols conjugated with branched polysaccharides rich in neutral sugars were obtained by PHWE. Meanwhile, oxidized polyphenol–polysaccharide structures were obtained by alkaline extraction. Polysaccharides–polyphenols conjugates showed an antidiabetic potential, although lower than free polyphenols. While polyphenols bound to polysaccharides with branched sidechains were able to attenuate glucose transport through Caco-2 human cell monolayers and showed a higher ability to reduce a-amylase activity, oxidized polyphenolic structures had a higher impact on a-glucosidase activity and on the antiglycation effect of the polysaccharide–polyphenol conjugates. Bearing this in mind, grape pomace polysaccharide–polyphenol conjugates may present a valid option in controlling blood glucose levels.

This work will contribute to the knowledge related to glucose transepithelial transport, enzyme and AGEs inhibition by polysaccharide–polyphenol conjugates. The understanding of the nutritional consequences of polysaccharide–polyphenol complexes in the colon and the convincing demonstration of the existence of covalent bonds between flavonoids and cell wall polysaccharides by structural elucidation of molecules are also fundamental aspects that should be addressed in the near future.

## Figures and Tables

**Table 1:** Chemical composition and antioxidant activity of free polyphenols and polysaccharide–polyphenol conjugates recovered from red grape pomace by solid–liquid extraction and pressurized hot water extraction. Results are presented as mean value ± standard deviation. For free polyphenols fractions or polysaccharide–polyphenol conjugates fractions, columns with the same symbol do not present statistical differences (*p* > 0.05).

Identification	FP	FP-95	AE	PHWE-95	PHWE-120
Yield (%)	1.5	1.3	2.6	1.3	3.4
Anthocyanins (μg Mv3Glc equi.mg^−1^)	121 ± 14 *	100 ± 13 *	-	-	-
Polymeric colour (%)	80.2 ± 0.2 *	30 ± 1 **	-	-	-
Non-anthocyanic compounds (μg GAE.mg^−1^)	30 ± 10 *	197± 13 **	-	-	-
Protein content (μg BSA equi.mg^−1^)	93 ± 3 *	105 ± 9 *	9 ± 2 *	10.4 ± 0.3 *	14.2 ± 0.5 **
Sugar content (μg Glucose equi.mg^−1^)	6.0 ± 0.3 *	6.7 ± 2 *	-	-	-
Phenolic compounds (μg GAE.mg^−1^)	254 ± 8 *	427 ± 24 **	106 ± 2 *	108 ± 5 *^,#^	111 ± 2 ^#^
Antioxidant activity (μM Trolox equi.)	2.6 ± 0.7 *	6.2 ± 0.3 **	15 ± 2 *	12 ± 2 *	16 ± 2 *
Antiradicalar activity (μM Trolox equi.)	13.7 ± 0.7 *	25 ± 2 **	16 ± 3 *	27 ± 1 **	25 ± 2 **

*, ** and ^#^ (*p* > 0.05)

**Table 2:** Yield, total carbohydrates, and monosaccharide composition (molar %) of polysaccharide–polyphenol conjugates (AE, PHWE-95, PWWE-120). Results are expressed as mean values ± standard deviation.

Sample	Carbohydrate Composition (Molar %)								Total Carbohydrates (mg.g^−1^)
	Rha	Fuc	Ara	Xyl	Man	Gal	Glc	GalA	
AE	1 ± 0	1 ± 0	9 ± 0	1 ± 0	1 ± 1	4 ± 1	4 ± 1	79 ± 3	687 ± 4
PHWE-95	1 ± 0	-	20 ± 2	2 ± 0	5 ± 0	8 ± 0	7 ± 1	59 ± 1	668 ± 43
PHWE-120	1 ± 0	-	2 ± 0	-	9 ± 0	4 ± 0	27 ± 1	58 ± 1	608 ± 4

**Table 3:** IC_50_ of α-Amylase and α-glucosidase inhibition by free polyphenols extracts (FP, FP-95) and polysaccharide–polyphenol extracts (AE, PHWE-95, PHWE-120). Results are presented as the mean ± standard error of the mean (SEM) (*n =* 9). Values with the symbol (*) are different from the positive control (*p* < 0.05). For the same enzyme, columns with different letter are significantly different (*p* < 0.05).

	IC_50_ ± SEM (µg.mL^−1^)					
	Positive Control	Free Polyphenols Extracts		Polysaccharide-Polyphenol Extracts		
	Acarbose	FP	FP-95	AE	PHWE-95	PHWE-120
α-amylase	2.5 ± 0.1	25 ± 1 *^,a^	27.5 ± 0.9 *^,a^	2139 ± 13 *^,b^	572 ± 22 *^,c^	939 ± 37 *^,d^
α-glucosidase	123 ± 67	0.48 ± 0.02 *^,a^	0.45 ± 0.02 *^,a^	2.7 ± 0.1 *^,b^	4.0 ± 0.2 *^,c^	5.2 ± 0.1 *^,d^

**Table 4:** IC_50_ of AGE inhibition by polyphenols extracts (FP and FP-95) and polysaccharide–polyphenols conjugates (AE, PHWE-95, PHWE-120). Results are presented as the mean ± standard error of the mean (SEM) (*n =* 9). Values with the symbol (*) are different from the positive control (*p* < 0.05). Columns with different letter are significantly different (*p* < 0.05).

IC_50_ ± SEM (μg.mL^−1^)					
Aminoguanidine	FP	FP-95	AE	PHWE-95	PHWE-120
230 ± 12	571 ± 32 *^,a^	209 ± 21 ^b^	1036 ± 79 *^,c^	1270 ± 65 *^,d^	5129 ± 594 *^,e^

## Data Availability

Not applicable.

## Supplementary Materials

The following are available online at https://www.mdpi.com/article/10.3390/nu13124495/s1, Table S1. HPLC−DAD /ESI-MS profile of anthocyanic compounds detected in free polyphenol fractions obtained from grape pomace (CFP and FP-95); Figure S1. HPLC−DAD /ESI-MS chromatogram of free polyphenols fractions (anthocyanic compounds) obtained from grape pomace; Table S2. HPLC−DAD /ESI-MS profile of nonanthocyanic compounds (low molecular weight phenolic compounds, flavan-3-ols and flavonols) detected in free polyphenol fractions obtained from grape pomace (CFP and FP-95); Figure S2. HPLC−DAD /ESI-MS chromatogram of free polyphenols fractions (non-anthocyanic compounds) obtained from grape pomace.
