# Supplementary material for: The Antidiabetic Effect of Grape Pomace Polysaccharide-Polyphenol Complexes
Source: Nutrients. 2021 Dec 15;13(12):4495. doi: 10.3390/nu13124495 (PMC8709276; doi:10.3390/nu13124495)
Supplement: Supplementary file 1 [file nutrients-13-04495-s001.zip › nutrients-1472123-supplementary.pdf]

The antidiabetic effect of grape pomace polysaccharide-polyphenol complexes

Filipa Campos[1], Andreia F. Peixoto[1], Pedro A.R. Fernandes[2], Manuel A. Coimbra[2], Nuno Mateus[1], Victor de Freitas[1], Iva Fernandes[1], Ana Fernandes[1,*]

[1] REQUIMTE – Laboratório Associado para a Química Verde, Departamento de Química e Bioquímica, Faculdade de Ciências, Universidade do Porto, Rua do Campo Alegre, 687, 4169-007 Porto, Portugal; filipasc96@hotmail.com; andreia.peixoto@fc.up.pt; nbmateus@fc.up.pt; vfreitas@fc.up.pt; iva.fernandes@fc.up.pt

[2] LAQV/REQUIMTE, Department of Chemistry, University of Aveiro, Campus Universitário de Santiago, 3810-193 Aveiro, Portugal; pedroantonio@ua.pt; mac@ua.pt;

*Correspondence: ana.fernandes@fc.up.pt; Tel.: +351220402596

Table S1. HPLC–DAD /ESI-MS profile of anthocyanic compounds detected in free polyphenol fractions obtained from grape pomace (CFP and FP-95).

Peak number	RT (min)	λ máx (nm)	$[M+H]^+$	MS^2	Tentative identification
1	35.00	523	465.6	303.1	Delphinidin-3-*O*-glucoside
2	42.19	526	479.9	317.1	Petunidin-3-*O*-glucoside
3	46.99	520	463.9	301.1	Peonidin-3-*O*-glucoside
4	48.59	526	493.6	331.7	Malvidin-3-*O*-glucoside
5	54.15	511	561.7	399.1	Carboxipyranomalvidin-3-O-glucoside
6	59.01	517	603.9	399.1	Carboxipyranomalvidin-3-O-acetylglucoside
7	60.71	529	521.8	317.1	Petunidin-3-*O*-acetylglucoside
8	66.87	529	535.9	331.1	Malvidin-3-*O*-acetylglucoside
9	69.30	529	611.8	303.2	Delphinidin-3-*O*-coumaroylglucoside
10	72.57	529	655.9	331.1	Malvidin-3-*O*-caffeoylglucoside
11	75.19	532	625.9	317.1	Petunidin-3-*O*-coumaroylglucoside
12	80.69	532	639.8	331.1	Malvidin-3-*O*-coumaroylglucoside

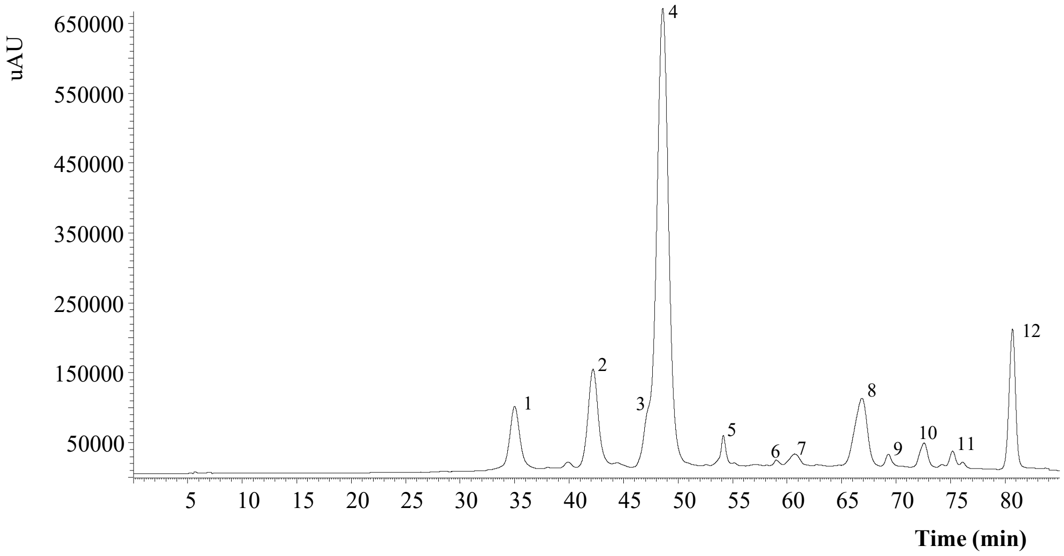

Figure S1. HPLC–DAD /ESI-MS chromatogram of free polyphenols fractions (anthocyanic compounds) obtained from grape pomace.

Table S2. HPLC–DAD /ESI-MS profile of nonanthocyanic compounds (low molecular weight phenolic compounds, flavan-3-ols and flavonols) detected in free polyphenol fractions obtained from grape pomace (CFP and FP-95).

Peak number	RT (min)	λ máx (nm)	$[M-H]^-$	MS^2	MS^3	Tentative identification
1	17.81	271	169.2	-	-	Gallic acid
2	21.11	277	865.1	695.0; 577; 425	524.93; 363.1; 289	Procyanidin trimer
3	23.52	292	593.2	467; 425; 407	-	(Epi)gallocatechin-(epi)catechin
4	33.35	277	897.1	729; 711; 593; 425	693; 543; 289	(Epi)gallocatechin-(epi)gallocatechin - (epi)catechin
5	37.58	277	881.1	729.0: 695.1; 577	303; 289	Procyanidin digalloylated dimer
6	38.11	277	1153.1	1026.9; 983; 865; 577	1010.1; 520.1	Procyanidin tetramer
7	39.48	280	577.3	425.1; 289.4	407.1; 273.1	Procyanidin dimer
8	42.04	280	577.3	425.1; 289.4	407.1; 273.1	Procyanidin dimer
9	46.11	280	865.2	695; 577	542.9; 541.1; 405	Procyanidin trimer
10	46.69	280	289.8	-	-	(Epi)catechin
11	48.20	280	865.4	695; 577	542.9; 541.1; 405	Procyanidin trimer
12	50.07	280	577.3	425.1; 289.4	407.1	Procyanidin dimer
13	52.07	277	1017.1	728,9	577.1	Procyanidin digalloylated trimer
14	53.52	289; 310	577.5	425.1	289.2; 407.1	Procyanidin dimer
15	54.81	283	1153.6	-	-	Procyanidin tetramer
16	57.32	277	881.4	695.1; 577; 425; 407.3	534.2; 425.1; 256.1	Procyanidin digalloylated dimer
17	58.41	289; 298; 307	325.1	-	-	Coumaric acid-*O*-glucoside
18	59.35	277	289.1	-	-	(Epi)catechin
19	60.70	280	729.5	577.3; 425.2; 407.3	451.2; 407.1; 289	Procyanidin galloylated dimer
20	61.71	280	865.4	695; 577.0; 425.1;	525.9; 677.1; 243.1	Procyanidin trimer
21	62.98	280	865.4	695; 577; 289	542.0; 677.1; 363.2	Procyanidin trimer

22	65.45	280	1153.4	983.9; 865.0	838.4; 609.1; 694.5	Procyanidin tetramer
23	66.73	277	729.4	407.3; 577; 289.3	285.1; 389.3	Procyanidin galloylated dimer
24	68.76	283	729.5 /865.2 / 1017.1	-	-	-
25	74.2	280	479.65	317.1: 271.2	271; 287.1; 179.2; 151	Myricetin-3-*O*-glucoside
26	77.95	280	1017.5	729	577	Procyanidin digalloylated trimer
27	79.54	280	865	695	543; 451	Procyanidin trimer
28	82.41	280	1017.3	729	407.2; 577	Procyanidin digalloylated trimer
29	88.98	352	463.5	301.1	179	Quercetin-3-*O*-hexoside
30	90.2	289; 349	477.5	301.2	179.17; 151.1	Quercetin-3-*O*-glucuronide
31	93.09	280	363.6	165.1; 196.9; 319.2	137.3	Unknown
32	98.04	-	449.1	303.1; 285.1;	285.1	Myricetin-3-*O*-arabinoside

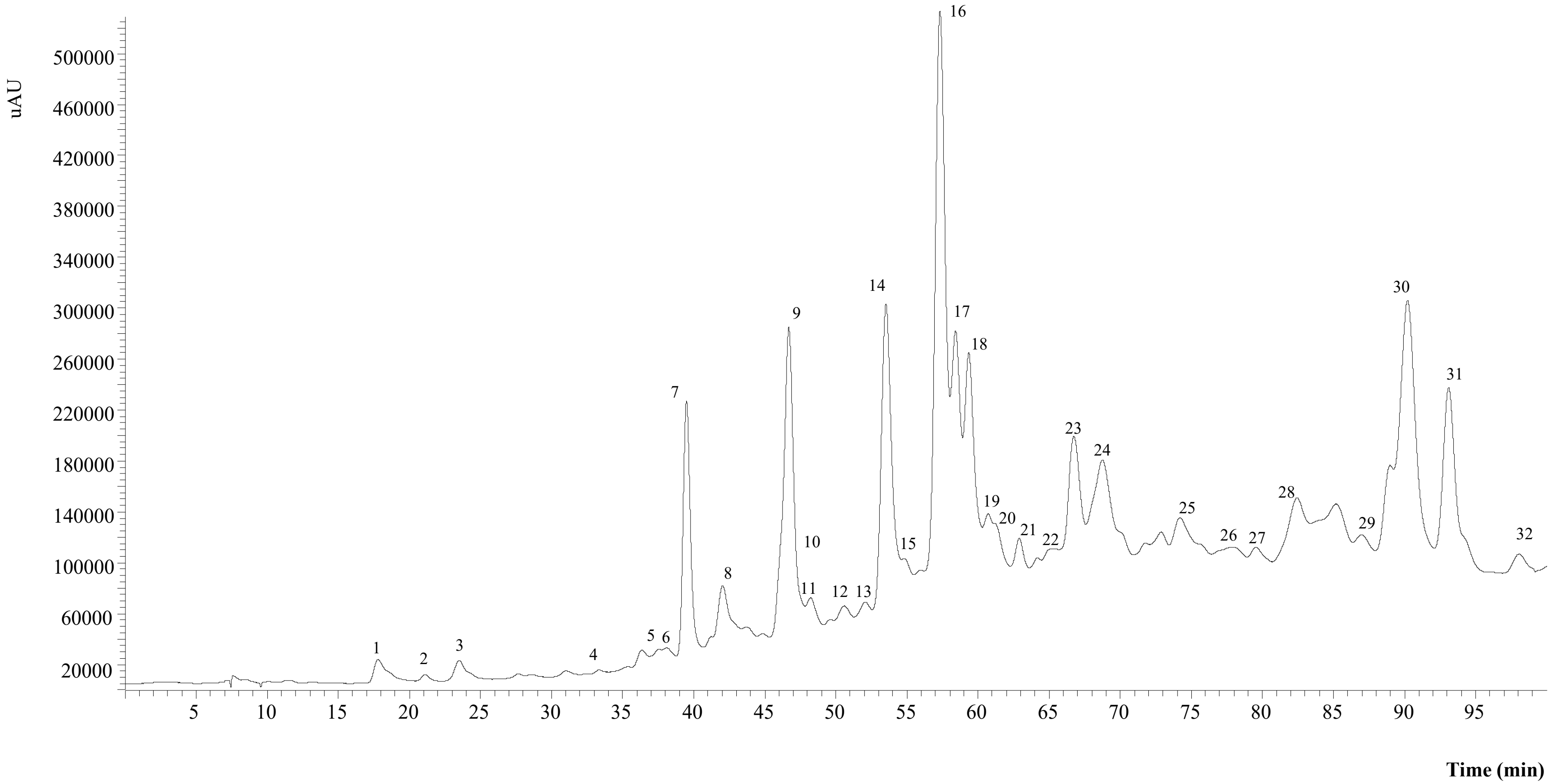

Figure S2. HPLC–DAD /ESI-MS chromatogram of free polyphenols fractions (non-anthocyanic compounds) obtained from grape pomace.
